# The Effect of Surface Roughness on the Contact Line and Splashing Dynamics of Impacting Droplets

**DOI:** 10.1038/s41598-019-51490-5

**Published:** 2019-10-21

**Authors:** Miguel A. Quetzeri-Santiago, Alfonso A. Castrejón-Pita, J. Rafael Castrejón-Pita

**Affiliations:** 1 Queen Mary University of London, School of Engineering and Materials Science, London, E1 4NS UK; 2 University of Oxford, Department of Engineering Science, Oxford, OX1 3PJ UK

**Keywords:** Fluid dynamics, Applied physics

## Abstract

Whether a droplet splashes upon impact onto a solid is known to depend not only on the fluid properties and its speed, but also on the substrate characteristics. Past research has shown that splashing is heavily influenced by the substrate roughness. Indeed, in this manuscript, we demonstrate that splashing is ruled by the surface roughness, the splashing ratio, and the dynamic contact angle. Experiments consist of water and ethanol droplets impacting onto solid substrates with varying degrees of roughness. High speed imaging is used to extract the dynamic contact angle as a function of the spreading speed for these impacting droplets. During the spreading phase, the dynamic contact angle achieves an asymptotic maximum value, which depends on the substrate roughness and the liquid properties. We found that this maximum dynamic contact angle, together with the liquid properties, the ratio of the peak to peak roughness and the surface feature mean width, determines the splashing to no-splashing threshold. In addition, these parameters consistently differentiate the splashing behaviour of impacts onto smooth hydrophilic, hydrophobic and superhydrophobic surfaces.

## Introduction

The impact of liquid droplets on solid dry substrates has been widely studied since the works of Worthington in 1895^1^. This simple process is important in many industrial situations, such as coating, inkjet printing and (aerospace) wing design^2–7^. Moreover, droplet impact is found in many natural phenomena, such as raindrop impact on oceans and sand or on tree leaves. Past research has found that the impact of a droplet on a smooth solid surface leads to one of at least five different outcomes, namely smooth deposition, splashing, partial or total rebound, and receding breakup^8^. The final outcomes depend on the liquid, solid, and surrounding air characteristics; the impact speed, and the size of the droplet^9,10^. Further studies on micro-structured surfaces, rigid metallic meshes and textiles have concluded that droplet penetration is determined by the structure’s pore size and the liquid and impacting properties^11–14^. Other studies have determined that the dynamics of droplet impact can be described by various dimensionless numbers. These include the Weber number ${\rm{We}}=\frac{\rho {D}_{0}{U}_{0}^{2}}{\sigma }$, the Reynolds number ${\rm{Re}}=\frac{\rho {D}_{0}{U}_{0}}{\mu }$, the Capillary number ${\rm{Ca}}=\frac{We}{Re}$, or the Ohnesorge number ${\rm{Oh}}=\frac{\mu }{\sqrt{\rho \sigma {D}_{0}}}$, where *U*_0_ is the impact velocity, *D*_0_ the diameter, and $\rho$, *μ* and *σ* are the droplet density, viscosity and surface tension, respectively. At low Weber and Reynolds numbers, a droplet spreads reaching a maximum diameter (*D*_*m*_), and then it undergoes a phase of receding and spreading until an equilibrium contact diameter is reached^15^. In fact, great interest exists in industry in predicting the maximum spreading diameter (or droplet footprint) in terms of the liquid and impacting properties. The droplet’s footprint controls several ‘quality factors’, such as printing resolution in inkjet technologies, and the coating efficiency in spray painting. Clanet *et al*. in 2004^16^ determined that the spreading diameter follows the scaling *D*_*m*_ ∝ We^1/4^ for both hydrophobic and hydrophilic smooth surfaces. This scaling was subsequently validated by experimental data for impacting droplets in the range of 2 ≤ We ≤ 900. Similarly, Laan *et al*. in 2014^17^ proposed a crossover between capillary and viscous effects obtaining the scaling ${D}_{m}\propto {{\rm{Re}}}^{1/5}\,f({\rm{We}}\,{{\rm{Re}}}^{-2/5})$. Moreover, Yunemoto and Kunigi in 2017 were able to predict *D*_*m*_ over a range of We and Re numbers by introducing the adhesion energy of the contact line^18^. Studies by Vadillo and Yokoi *et al*. in 2009 concluded that the maximum spreading diameter is affected by the dynamic contact angle, which is in turn influenced by the substrate wettability and the viscosity of the fluid^19,20^.

Recent studies focusing on the dynamic contact angle on rough substrates found that the maximum spreading diameter depends on the Weber number and the contact angle at maximum spreading^21^. Roughness affects the spreading of droplets on hydrophilic grains^22^, but has a minor effect on the spreading of water drops impacting hydrophobic grains^22^. Moreover, Bayer and Megaridis in 2006 found that the slip length, i.e. the scale where the no-slip boundary condition ceases to be valid, and the contact line dynamics are both influenced by substrate roughness^15^. In grooved surfaces, the pinning of the drop at an edge of a substrate structure causes the contact angle to vary (increase) thus affecting spreading^23^.

Splashing is defined as the process in which a sheet of liquid ejected upon impact breaks up into droplets. This disintegration of the drop is often classified into two main categories: corona and prompt splashing. In the former, the liquid sheet that emerges at the front of the advancing contact line is lifted above the substrate and eventually breaks up creating secondary droplets. In the latter, secondary droplets are rapidly ejected parallel to the substrate from the advancing lamella soon after impact. There is no formal definition of corona and prompt splashing apart from that based on the observable shape and timing of splashing, i.e. prompt splashing occurs quickly after impact and often at an angle parallel to the substrate, and corona splashing results from the fragmentation of the formed lamella^10^. However, as observed by Latka *et al*. in 2012, both types of splashing can occur within the same We and Re numbers^24^. In contrast, in 2015, Roisman, Lembach and Tropea demonstrated that prompt splashing on smooth substrates is found at high Reynolds and Weber numbers, i.e. Re > 4,000 and We > 400^25^. Liquid, surface and the surrounding air properties are known to affect splashing^24–30^. Previous studies have shown that the air dynamics around an impacting droplet and a substrate can control the splashing outcome. In particular, it is known that the air dynamics near the impact point do not influence splashing, but it is the air around the spreading edge that does it^31^. Moreover, in a recent work at high Weber and Reynolds numbers, Burzynski & Bansmor concluded that splashing is not caused by gas entrainment on glass substrates^32^. However, Tsai *et al*. in 2010, using micro-textured substrates, demonstrated that the “trapped air pocket” can escape through the micro-structures forming the substrate, thus effectively controlling splashing^33^. In addition, Latka *et al*. in 2012 found that substrate roughness and liquid viscosity can suppress thin-sheet splashing and control prompt splashing^24^. In 2015, Roisman, Lembach and Tropea determined that the ambient pressure does not determine splashing for ethanol and water droplets impacting rough and porous substrates^25^. Finally, Langley *et al*. in 2018 found that nanometric roughness causes the entrainment of microbubbles at the edge of the lamella^34^. Moreover, the substrate roughness is a parameter known to influence the dynamics upon impact where even a single protrusion on a substrate’s surface can trigger splashing^25,35–38^. Hao in 2017 found that corona splashing is affected by surface tension and can be suppressed by substrate roughness^36^. In fact, surface roughness can suppress corona splashing and promote prompt splashing^8,24,39^.

The effect of roughness on the splashing behaviour has been studied in terms of various parameters, including the arithmetic amplitude average roughness (R_*a*_), the root-mean-square roughness (R_*rms*_), the average peak to peak feature size (R_*pk*_) and the surface feature mean width (R_*sm*_) defined as the width average of the profile features^24,25,35–37^. In 1998, Range and Feuillebois^35^ proposed that splashing was determined by the We number and the ratio between the average surface roughness ratio and the droplet size, i.e. *R*_*a*_/*D*_0_. In contrast, other studies have suggested that the scalings (We/Oh)^1/2^ and the ratio *D*_0_/*R*_*a*_ can separate splashing from no-splashing events^37^. However, Roisman *et al*. in 2015 argued that the mean average roughness alone does not provide sufficient information of the substrate surface to effectively characterise the splashing threshold^25^. They found that the characteristic slope of the protruding peaks, R_*pk*_/R_*sm*_, can characterise the splashing behaviour on rough and porous substrates. Accordingly, further studies with micro-textured substrates have been focused on identifying the influence of other surface-related geometrical parameters on droplet splashing. For instance, in 2018, Zhang *et al*., used an array of equally sized cylindric pillars and determined that both the pillars height and spacing (or pillar density) are relevant parameters affecting splashing^40^. Additional experiments with multi-scale carbon nano-fiber substrates show that, for We < 120, micrometer-scale substrate structures promote splashing but nano-scale features play a minor role on splashing^41^.

The substrate roughness is also known to influence wettability and the contact angle of droplets resting on a substrate^42–44^. In 1936, Wenzel^43^ found that the static contact angle depends on the ratio between the actual wetted area and the droplet projected area; this ratio is called the roughness factor. Later, Cassie and Baxter in 1944^44^ found that droplets can remain suspended on an air cushion created by the substrate roughness. Finally, very recently (2019), Quetzeri-Santiago *et al*. determined that surface wettability plays a crucial role over the splashing behaviour, as it controls the dynamic contact angle of the rapidly spreading liquid front formed upon drop impact^45^. All this evidence leads to the hypothesis that surface roughness effectively affects the dynamic contact angle, which in turn governs splashing.

In this work, we have used high speed imaging to visualise the impact of droplets on optical glass diffusers of different roughnesses and wetting properties to determine the role of the substrate roughness on the impact dynamics. Wettability, described through the dynamic contact angle, is studied in terms of the spreading speed. Surface roughness is characterised via atomic force microscopy (AFM) and surface profilometry. Our results indicate that micrometer- and nanometer- scale roughness actively affects the dynamic contact angle and consequently the splashing behaviour. We conclude by establishing that the splashing behaviour is effectively characterised by three parameters, i.e. the splashing ratio, the feature roughness size ratio, and the maximum dynamic contact angle.

## Methods

**Figure 1:**
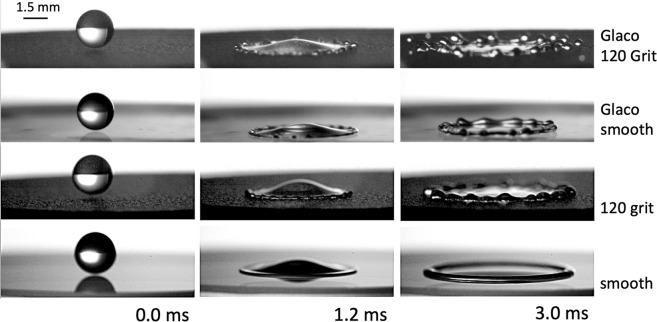
Snapshot sequences of water droplets impacting on Glaco-sprayed on 120-grit glass (Movie 1 in the Supplementary Material), Glaco-sprayed on smooth glass (Movie 2 in the Supplementary Material), 120-grit rough glass, and smooth (uncoated) glass at ${U}_{0}=2.05\,{\rm{m}}/{\rm{s}}$. Splashing is only observed on the Glaco-sprayed 120-grit rough glass.

**Figure 2:**
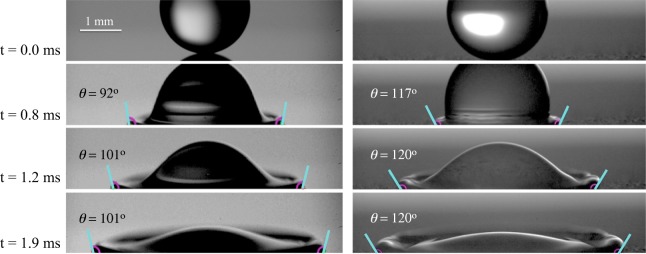
Illustration of the image analysis of a water droplet spreading on smooth (left) and 220 grit (right) diffusers. The blue lines show the tangent to the droplet at the pinning point (contact angle).

Ethanol and water liquid droplets were produced by dripping and allowed to impact N-BK7 glass substrates of various defined roughnesses at atmospheric pressure, examples are seen in Figs 1 and 2. Droplets were generated by a 1.0 mm diameter needle attached to a syringe pump (Razel, model R99-E) operating at a flow rate of 1.94 mm^3^/s. We have assumed that the droplet diameter remained constant during experiments. A recent work by Niimura and Hasegawaka in 2019 demonstrated that similarly sized (*D*_0_ = 1.4 mm) ethanol droplets, levitated in air at ambient temperature, initially evaporate at a rate of 2.5% in 5 seconds^46^. Assuming this evaporation rate, our 1.9 mm diameter droplet would reduce its diameter by 0.3 *μ*m in the 3.0 ms duration of our experiments. This is below the resolution of our optical system and its effect negligible in the experimental error. The impact speed was controlled and varied by adjusting the height of droplet release. The drop diameter (*D*_0_) was kept constant at 1.9 ± 0.1 mm for ethanol and at 2.4 ± 0.1 mm for water. Impact events were recorded by a high-speed camera (Phantom V711) coupled to a 12x UltraZoom Navitar lens with a 1.5x tube adapter. The illumination was performed by a 100 W LED array. A 100 × 100 m^2^ glass diffuser from Comar Optics was placed between the substrate and the LED array to obtain an even background. The resolution was set at 1,280 × 256 pixels^2^ with a recording speed of 23,004 frames per second, at an exposure time of 10.0 *μ*s. The effective resolution was 16.39 *μ*m per pixel. Impact speeds and droplet diameters were measured by image analysis (Matlab).

**Figure 3:**
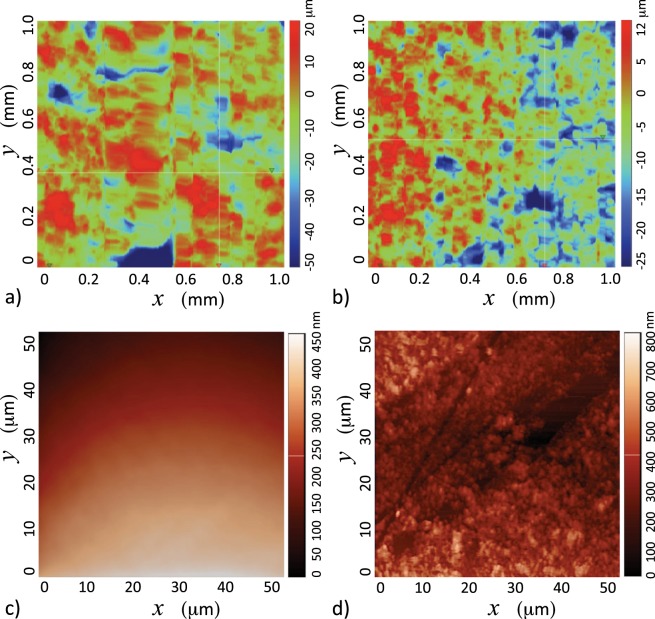
Surface roughness as seen by profilometry for (**a**) 120 grit, and (**b**) 220 grit substrates. Atomic force microscopy of (**c**) smooth glass, and (**d**) Glaco-sprayed on smooth glass.

Four flat N-BK7 uncoated ground glass diffusers and an optical window (of the same material) from Thorlabs were used as impacting surfaces. Additional experiments were performed by spraying these substrates with a superhydrophobic coating (Glaco). The roughness of these samples was characterised either by profilometry or by atomic force microscopy (AFM). The ground diffusers had reported roughnesses of 120, 220, 600 and 1,500 grit. The roughness of the glass diffusers was measured with a tactile surface profilometer (DektakXT Stylus Profiler) in 3 areas of 1.0 mm^2^ each, taking 5,000 data points per scan. Both the smooth uncoated and the Glaco-sprayed optical windows were characterised by AFM (NT-MDT NTEGRA) in the semi-contact mode topography. AFM scans were conducted at a frequency of 1.01 Hz on 3 different zone samples of 50 × 50 *μ*m^2^ each. Example profilometry measurements are shown in Fig. 3a,b, and AFM results are shown in Fig. 3c,d. The average roughness (R_*a*_), the peak to peak roughness (R_*pk*_) and the mean width of the surface features R_*sm*_ of the surfaces are reported in Table 1. We have reported the mean width of the roughness feature of the smooth glass as R_*sm*_ > 50 *μ*m, as this value corresponds to the size of the AFM sample size (no features were found within).

**Table 1:** Surface roughness for the substrates used throughout the experiments.

Substrate	R_*a*_ (*μ*m)	R_*pk*_ (*μ*m)	R_*sm*_ (*μ*m)
Smooth Glass	0.017	0.04	>50
1500 Grit	1.021	2.39	40.32
600 Grit	1.028	2.48	41.99
220 Grit	3.507	11.65	152.03
120 Grit	5.743	20.02	168.49
Glaco Glass	0.422	0.82	25.58
Glaco 1500 Grit	1.064	2.41	40.47
Glaco 220 Grit	3.618	11.43	151.95
Glaco 120 Grit	5.807	20.08	168.27

In our experiments, the onset of splashing occurs in the range of *U*_0_ ≈ 1.5 to 1.8 m/s for ethanol and water. Consequently, we measured the dynamic contact angle at 1.01 m/s because, at this condition, impacting events result on simple spreading where the contact line is free from instabilities. The contact angle was measured by image analysis from each frame of the high-speed recording, and this is illustrated in Fig. 2. Image analyses, to extract the contact angle from the high-speed images, were carried out by an in-house developed routine in Matlab^45^ and ImageJ. In these spreading experiments, the effective resolution of our optical systems is of 6.47 *μ*m per pixel. In Matlab, the interface was reconstructed using a second-order polynomial regression applied over the droplet boundary within a region of 25 pixels from the contact line. The Matlab code was tested and verified by measurements done in ImageJ. The camera axis was angled downward by 2 degrees to obtain a clear view of the contact line. The projected height (*h*′) of the section of the droplet, used to determine the contact angle, depends on the tilting angle ($\varphi$), as $h^{\prime} =hcos(\varphi )$, where *h* is the true height. Consequently, a tilting angle of 2 degrees affects our contact angle measurement by 0.6%.

The static advancing and receding contact angles were measured following the method developed by Kwok *et al*. in 1996^47^. In our experimental setup, through a syringe tip, an infuse/withdraw syringe pump (Harvard Apparatus PHD 4400) expands or contracts a droplet over a substrate at a rate of 12 *μ*l/min. The process was recorded at 200 fps with the same spatial resolution as the impacting experiments. Results are shown in Table 2. Parametric experiments were carried out with water and ethanol droplets by varying the droplet impact speed to include conditions of spreading and splashing. As explained in the introduction, in this work, splashing refers to conditions where the rim of the spreading droplet breaks into secondary droplets. Each experiment was conducted three times using clean and dust-free surfaces. We identified and characterised spreading and splashing conditions for all the substrate/fluid combinations. In our conditions, droplet impact speeds range from 1.38 to 2.98 m/s, 63.48 < We < 548 and 1790 < Re < 7,152.

**Table 2:** Dynamic contact angles for the substrates used throughout the experiments.

Substrate	*θ*_*max*_ Water (deg)	*θ*_*adv*_ Water(deg)	*θ*_*rec*_ Water(deg)	*θ*_*max*_ Ethanol (deg)	*θ*_*adv*_ Ethanol (deg)	*θ*_*rec*_ Ethanol (deg)
Smooth Glass	101 ± 3	92 ± 3	70 ± 3	97 ± 5	20 ± 3	0 ± 3
1500 Grit	112 ± 3	89 ± 3	26 ± 3	97 ± 5	11 ± 3	0 ± 3
600 Grit	113 ± 3	—	—	97 ± 5	—	—
220 Grit	120 ± 5	95 ± 3	22 ± 3	101 ± 5	9 ± 3	0 ± 3
120 Grit	129 ± 5	98 ± 3	27 ± 3	107 ± 5	8 ± 3	0 ± 3
Glaco Glass	149 ± 3	161 ± 3	158 ± 3	—	—	—
Glaco 1500 Grit	149 ± 3	161 ± 3	159 ± 3	—	—	—
Glaco 220 Grit	150 ± 5	160 ± 3	156 ± 3	—	—	
Glaco 120 Grit	150 ± 5	158 ± 3	156 ± 3	—	—	—

## Results and Discussion

The AFM analysis indicates that the superhydrophobic coating (Glaco) does not significantly affect the (micrometer sized) roughness (except for the smooth optical window), as shown in Table 1. These tests proved that the roughness of the diffusers is found in the micrometer range and that the application of Glaco only alters the roughness at the tenths of nanometers scale. The exception to this rule is the optical window (smooth glass) where the Glaco coating increases the roughness parameter R_*a*_ by ≈400 nm. The experimental conditions thus include surface features in the range from tents of nanometers (smooth glass) to the tents of micrometers (120 grit diffuser).

In our experiments, we found that the maximum spreading diameter of a droplet impacting at 1.01 m/s is 6% smaller on the 120-grit surface than that on the smooth substrate. In contrast, for the Glaco covered substrates, the maximum spreading diameter difference between the 120 grit and the smooth surfaces is of only 1%. This is consistent with past works that demonstrated that roughness has a small effect on the maximum spreading at high We and Re numbers^48,49^. Other works, also in agreement with our findings, have demonstrated that spreading is more affected by roughness on hydrophilic substrates than on hydrophobic ones^23,50,51^. At U_0_ = 2.05 m/s, the smallest spreading diameter was found on the Glaco covered smooth glass (D_*m*_ = 2.87), while the (uncoated) smooth glass showed the largest spreading diameter (D_*m*_ = 3.59). Under the same conditions, a droplet impacting the 120 grit substrate produced a maximum spreading diameter 3% smaller than that for the smooth substrate. This observation is also consistent with previous works that found that roughness hinders the spreading of impacting drops^37^.

**Figure 4:**
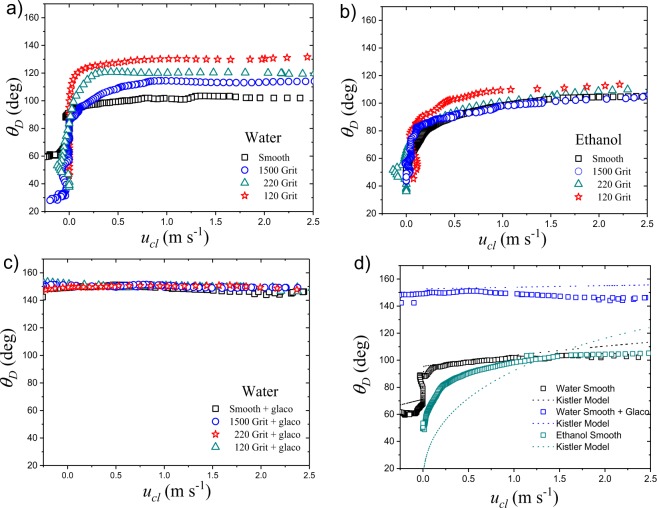
The dynamic contact angle *θ*_*D*_ in terms of the contact line speed *u*_*cl*_ for ethanol and water spreading on substrates of different roughness. Here, the figures shows the impact of (**a**) water droplets on uncoated substrates, (**b**) ethanol on uncoated substrates, (**c**) water droplets on Glaco coated substrates, and (**d**) shows the comparison between the experimental dynamic contact angle and appropriate Kistler Model for smooth substrates.

The dynamic contact angle measurements in terms of the contact line speed for water and ethanol droplets, impacting different substrates, are found in Fig. 4. In all the cases studied here, the dynamic contact angle presents a characteristic (asymptotic) maximum value (*θ*_*max*_), at spreading speeds in the range 2.5 > *u*_*cl*_ > 0.25 m/s. These results are consistent with past experiments carried out on smooth flat surfaces^19,20,45^. Consequently, it is interesting to note that droplets spread at a constant dynamic contact angle over rough substrates too.

Given the differences on the maximum dynamic contact angle found between water and ethanol droplets on the rough substrates (Fig. 4), these results firmly indicate that the contact angle is influenced by both the surface roughness and the liquid properties. Within our experimental conditions, this effect is most visible in water impacting the N-BK7 glass surfaces, where the maximum dynamic contact angle varies from 101 degrees on the smooth substrate to 129 degrees on the 120-grit (roughest) surface. For ethanol droplets, the influence of the substrate roughness on the dynamic contact angle also follows this trend; within standard errors, the dynamic contact angle increases as the roughness increases. Additionally, our results indicate that Glaco-coated substrates (superhydrophobic) acquire the same *θ*_*max*_ regardless of the micrometer sized roughness. In agreement with a past work^45^, the condition ${\theta }_{max} > 97$ degrees is found for all the conditions explored here. In fact, within our conditions, an upper limit for the maximum dynamic contact angle was found at ${\theta }_{max}=150$ degrees.

Ethanol and water droplets were used in our experiments to provide two systems with similar densities (${\rho }_{water}=998\,{\rm{kg}}/{{\rm{m}}}^{3}$ and ${\rho }_{ethanol}=780\,{\rm{kg}}/{{\rm{m}}}^{3}$) and viscosities (${\mu }_{water}=0.98\,{\rm{mPa}}\,{\rm{s}}$ and ${\mu }_{ethanol}=1.04\,{\rm{mPa}}\,{\rm{s}}$) but very different surface tensions (${\sigma }_{water}=70.8\,{\rm{mN}}/{\rm{m}}$ vs ${\sigma }_{ethanol}=22.3\,{\rm{mN}}/{\rm{m}}$). In this way, capillary effects are differentiated from inertia, and thus any difference in the dynamic contact angle between water and ethanol can be solely associated to a surface tension disparity. Our results reveal that the low surface tension of ethanol facilitates spreading and is less affected by roughness. In fact, this is consistent with previous works where roughness had a limited effect on low-surface tension droplets spreading and splashing over wettable substrates^21,36,52^.

According to the empirical relationship of Kistler^53^, the dynamic contact angle is determined by the liquid properties and the equilibrium contact angle. We found that the Kistler model consistently underestimates the maximum advancing contact angle for rough substrates. In contrast, as seen in Fig. 4d, the model fits reasonably well the experiments with water on smooth substrates if the static advancing contact angle is used instead of the equilibrium contact angle. The discrepancies seen on the dynamics of ethanol, Fig. 4d, can be attributed to the large difference found between the static advancing contact angle and the maximum dynamic contact angle. In fact, one of the model’s assumptions is that static and dynamic contact angles are similar at low speeds, but from the data in Table 2, we see this condition is not satisfied.

The splashing ratio *β* includes both the liquid and impacting properties, and has been successfully used to characterise splashing on smooth wettable substrates^26,52^. Additionally, a recent work has determined that the splashing ratio, and the maximum dynamic contact angle *θ*_*max*_, are sufficient to separate the splashing and no-splashing behaviour of impacting droplets on hydrophilic, hydrophobic and superhydrophobic surfaces^45^. The splashing ratio is given by

1 $$\beta \propto \frac{{\mu }_{g}^{1/2}{(\rho D{U}_{0}^{5})}^{1/6}}{{\sigma }^{2/3}},$$

where *μ*_*g*_ is the viscosity of air. Under the parametrisation $\beta \propto \varphi ({\theta }_{max})$ used by Quetzeri *et al*.^45^ surface roughness effects on the spreading and splashing are assumed to directly affect the dynamic contact angle. Equation 1 is independent from viscosity but we expect viscosity to play a significant role in splashing in line with previous works^24,54^. Our premise is that viscosity influences the dynamic contact angle, as proposed before^45^. Figure 1 presents the dynamics of a water droplet impacting various substrates with different roughness and different wettabilities (all at a speed of 2.05 m/s). Experiments show that splashing is observed for the 120 grit Glaco-sprayed substrate, but not on the smooth Glaco-sprayed surface, demonstrating that two identical droplets impacting with the same speed a substrate of the same wettability (same *θ*_*max*_), but different surface roughness can have a different splashing outcome. Under our experimental conditions, water droplets prompt splashed on all the substrates but corona splashing is seen with ethanol droplets impacting the smooth (Movie 3 in the Supplementary Material) and the 1,500 grit substrates. In agreement with past works, we observed that surface roughness can suppress corona splashing and promote prompt splashing^8,24,39^. At the same impacting speeds, e.g. *U*_0_ = 2.24 m/s, ethanol droplets present corona splashing on the 1,500 grit substrate but prompt splashing on the 120 grit surface.

**Figure 5:**
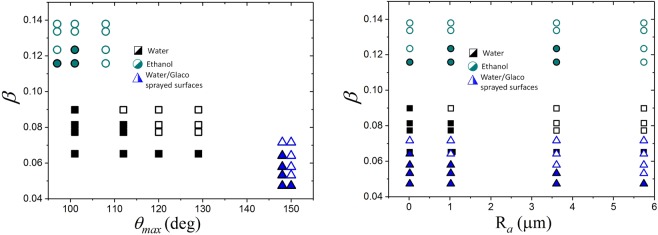
Left; splashing behaviour in terms of splashing parameter *β* and the maximum dynamic contact angle *θ*_*max*_. Right; splashing behaviour in terms of splashing parameter *β* and the arithmetic amplitude average roughness (*R*_*a*_). Open symbols represent splashing while solid ones stand for no splashing.

Figure 5a shows that *β* and *θ*_*max*_ are not sufficient to well-divide the splashing behaviour for the all the substrates. Previous studies have attempted to capture the roughness contribution to splashing through the average surface roughness R_*a*_^35,37^. However, as seen in Fig. 5b, the splashing ratio *β* and R_*a*_ are also not able to separate the splashing behaviour. We argue that *θ*_*max*_ or R_*a*_ do not adequatly characterise splashing because, as noted by previous research^41^, wetting is determined by the multi-scale nature of the roughness features. Depending on the liquid and impacting properties, a droplet might spread following one of the various states described by Belaud *et al*. in 2015^55^. Four states named Wenzel, Cassie-Baxter, Cassie-Baxter-Wenzel, and Wenzel-Cassie-Baxter have been identified. In the Wenzel state, a spreading droplet would wet all the features of the substrate. In other states, namely Cassie-Baxter, Cassie-Baxter-Wenzel, and Wenzel-Cassie-Baxter, a droplet would spread over the substrate, without wetting either or none of the nanometer or the micrometer sized features. In the Wenzel-Cassie-Baxter state, a liquid would penetrate the nanometer features without wetting the micrometer roughness^55^. In contrast, a droplet would penetrate the micrometer sized features without wetting the nanometer structures in the Cassie-Baxter-Wenzel state. Finally, a Cassie-Baxter state would see the droplet spreading over a thin film of air without wetting any of the nanometer or micrometer structures. Our experiments cover a broad range of impact speeds and liquid properties; consequently, we expect to find some of these spreading states within our conditions. Indeed, spreading under Wenzel-Cassie-Baxter or Cassie-Baxter states provides an explanation as to why the micrometer roughness does not affect *θ*_*max*_ for the Glaco-covered surfaces, as shown in Fig. 4c. Under these scenarios, an impacting droplet would spread without wetting the micrometer sized features. According to Reyssat *et al*. all our spreading experiments are in the Cassie-Baxter state as the impact velocities are below the transitional critical impact speed to the Wenzel state (*u*_*c*_ = 3 m/s)^56^. In contrast, we claim that Wenzel-Cassie-Baxter states are not found in our Glaco-coated surfaces because the micro-sized structure is wettable and yet we observe bouncing. This is also reflected on the dynamic contact angle. The Glaco-coated smooth glass is in a Casie-Baxter state as there is no micro-sized structure. Furthermore, all the Glaco-coated substrates have the same dynamic contact angle indicating that all share the same state, i.e. Cassie-Baxter. At higher impacting speeds, a droplet could wet the micrometer structure and spread following a Cassie-Baxter-Wenzel state. The concept of multi-scale roughness was first developed by Roisman *et al*.^25^ in 2015 by introducing the parameter R_*pk*_/R_*sm*_ to characterise splashing on substrates with a random roughness. We argue that this surface parameter, additional to the contact angle *θ*_*max*_ and the splashing ratio *β*, is required to characterise splashing on rough surfaces. Following the approach of Wenzel^43^, we have used the cosine of the maximum advancing contact angle to obtain a dimensionless parameter for the wettability factor. Additionally, to resemble the trend of a previous work on smooth substrates^45^, we have introduced the factor (1-cos *θ*_*max*_). Furthermore, following the approach of Wenzel^43^, the surface roughness factor was multiplied by the wettability parameter. Finally, the additive factor (1 + R_*pk*_/R_*sm*_) is introduced to avoid zeroing the contribution of smooth substrates (R_*pk*_/R_*sm*_ is zero for smooth substrates).

**Figure 6:**
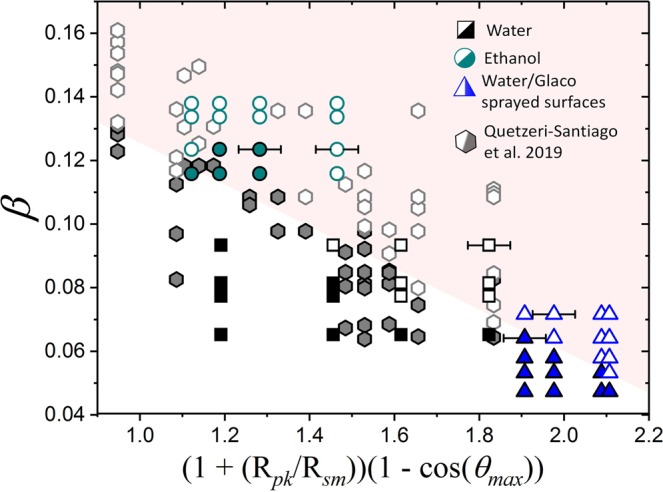
Splashing behaviour of water and ethanol drops in terms of the parameter (R_*pk*_/R_*sm*_)cos(*θ*_*max*_). The splashing behaviour is well characterised for the different roughness and wettabilities. Open symbols represent splashing while solid ones stand for no splashing. Example error bars are shown at selected points. The shadowed region is a guide for the eye to separate splashing from spreading.

Figure 6 shows our experimental data when parametrised by *β*, R_*pk*_/R_*sm*_ and *θ*_*max*_. As observed, these parameters successfully divide splashing and no splashing events for all rough and smooth substrates and liquids. Moreover, these parameters also separate the splashing behaviour of the experiments performed by Quetzeri *et al*. in 2019^45^, examples of the splashing and spreading of water droplets at high speeds are seen in Movies 4 (spreading on smooth glass) and 5 (splashing on Glaco-sprayed smooth glass) of the *Supplementary Material*. The research materials supporting this publication can be accessed in the Supplementary Information.

## Conclusions

In this manuscript, we have shown that substrate roughness affects the dynamic contact angle, and, as a result, the splashing of droplets impacting on solid surfaces. Our experiments demonstrate that an increase of surface roughness at the micrometer scale results in an increase of the maximum dynamic contact angle *θ*_*max*_. In addition, this effect depends on the liquid surface tension, as roughness affects the dynamic contact angle of water more than it affects ethanol. In contrast, we have observed that micrometer scale roughness does not affect the spreading dynamics on (nanometer induced) superhydrophobic surfaces. This observation is consistent with spreading occurring under a Cassie-Baxter state, where droplets do not wet the micrometer structures. These effects are included in our parametrisation by introducing the surface characteristics as the roughness ratio (peak to peak feature size over the surface feature mean width, R_*pk*_/R_*sm*_). We show that the splashing ratio *β*, combined with the maximum dynamic contact angle *θ*_*max*_, and the roughness ratio successfully characterises the splashing behaviour on the substrates used in this work. Our results also parametrise past experiments on smooth hydrophilic, hydrophobic and superhydrophobic surfaces.

## Supplementary information

Movie 1

Movie 2

Movie 3

Movie 4

Movie 5

Supplementary Dataset 1
